# The Immune-Related Gene HCST as a Novel Biomarker for the Diagnosis and Prognosis of Clear Cell Renal Cell Carcinoma

**DOI:** 10.3389/fonc.2021.630706

**Published:** 2021-04-23

**Authors:** Yongying Zhou, Xiao Wang, Weibing Zhang, Huiyong Liu, Daoquan Liu, Ping Chen, Deqiang Xu, Jianmin Liu, Yan Li, Guang Zeng, Mingzhou Li, Zhonghua Wu, Yingao Zhang, Xinghuan Wang, Michael E. DiSanto, Xinhua Zhang

**Affiliations:** ^1^Department of Urology, Zhongnan Hospital of Wuhan University, Wuhan, China; ^2^Department of Rehabilitation Medicine, Renmin Hospital of Wuhan University, Wuhan, China; ^3^Department of Urology, Huanggang Central Hospital, Huanggang, China; ^4^Department of Surgery and Biomedical Sciences, Cooper Medical School of Rowan University, Camden, NJ, United States

**Keywords:** prognosis, biomarker, clear cell renal cell carcinoma, HCST, immune-related gene

## Abstract

Clear cell renal cell carcinoma (ccRCC) is the most common type of kidney tumor worldwide. Analysis of The Cancer Genome Atlas (TCGA) and Gene Expression Omnibus (GEO) databases showed that the immune-related gene (IRG) hematopoietic cell signal transducer (HCST) could provide guidance for the diagnosis, prognosis, and treatment of ccRCC. The RNA-seq data of ccRCC tissues were extracted from two databases: TCGA (https://www.cancer.gov/about-nci/organization/ccg/research/structural-genomics/tcga) and GEO (https://www.ncbi.nlm.nih.gov/geo/). Corresponding clinical information was downloaded from TCGA. Immune-related gene data were extracted from the IMMPORT website (https://www.immport.org/). Differential analysis with R software (https://www.r-project.org/) was used to obtain a prognosis model of ccRCC IRGs. The differences were combined with the clinical data to assess the usefulness of the HCST as a prognostic biomarker. Based on data obtained from the Oncomine (https://www.oncomine.org/), Human Protein Atlas (https://www.proteinatlas.org/), and PubMed (https://pubmed.ncbi.nlm.nih.gov/) databases, the expression levels of the HCST in ccRCC, clinical-pathological indicators of relevance, and influence on prognosis were analyzed. Regulation of the HCST gene in ccRCC was assessed by gene set enrichment analysis (GSEA). In TCGA/GEO databases, the high HCST expression in tumor tissues was significantly correlated to the TMN stage, tumor grade, invasion depth, and lymphatic metastasis (*p* < 0.05). The overall survival (OS) of patients with high HCST gene expression was significantly lower than that of patients with low HCST gene expression (*p* < 0.001). Multivariate Cox regression analysis suggested that the HCST expression level [hazard ratio (HR) = 1.630, 95% confidence interval (CI) = 1.042–2.552], tumor cell grade (HR = 1.829, 95% CI = 1.115–3.001), and distant metastasis (HR = 2.634, 95%, CI = 1.562–4.442) were independent risk factors affecting the OS of ccRCC patients (all, *p* < 0.05). The GSEA study showed that there was significant enrichment in cell adhesion, tumorigenesis, and immune and inflammatory responses in HCST high expression samples. Hematopoietic cell signal transducer expression was closely associated with the levels of infiltrating immune cells around ccRCC tissues, especially dendritic cells (DCs). In conclusion, the present study suggested that the HCST was interrelated to the clinicopathology and poor prognosis of ccRCC. High HCST expression was also closely correlated with the levels of tumor-infiltrating immune cells, especially DCs.

## Introduction

Renal carcinoma is one of the most common malignant tumors of the urinary system and accounts for 3% of all adult cancers. Clear cell renal cell carcinoma (ccRCC) is the most common pathological type of renal carcinoma, accounting for 70–85% of all cases (1). However, non-surgical treatments for ccRCC, such as chemotherapy and radiotherapy, are limited due to uncertain efficacy, heavy patient burden, frequent side effects, and poor prognosis. More effective treatments with fewer side effects have been actively sought (2). Indeed, target therapy and immunotherapy have recently become as first-line therapies for ccRCC (3, 4).

Since the last century, bacillus Calmette–Guerin vaccine, interferon-alpha, and interleukin-2 (IL-2) have been used for immunotherapy of cancer. The application of IL-2 in tumor therapy has confirmed the effectiveness of adaptive immunity for cancer control and revealed T-cell regulation as a new strategy for immunotherapy. In fact, chimeric antigen receptor-modified T cells and immune modulation using antibodies to block immune regulatory checkpoints were named as the “breakthrough of the year” by *Science* in 2013 (5). Currently, with an unprecedented sustained and stable antitumor response, immunotherapy cytotoxic T lymphocyte-associated antigen 4 (CTLA4) or programmed cell death protein 1 (PD-1)/PD-1 ligand 1 (PD-L1) has demonstrated remarkable efficacy against various types of cancer (6).

Previous studies have reported that ccRCC is prone to immune cell infiltration and, thus, is highly responsive to immunotherapies that inhibit the interactions between immune cells and tumor cells by targeting CTLA4, PD-1, and PD-L1 (2). The blood, immune cells, and stromal cells surrounding cancer tissue form an immune microenvironment containing receptor factors involved in immunosuppression tolerance (7). Other studies have found that some indicators in the ccRCC microenvironment, such as CD8+T-cell density and PD-1/PD-L1 expression in the tumor and invasive margin (8), can be used as indicators to evaluate the clinical effectiveness of PD-1 inhibitors (9, 10). Hence, the identification of molecules as biomarkers that regulate the immune microenvironment is crucial to improving immunotherapy against ccRCC (11–13).

In the present study, analysis of public datasets identified 2,498 immune-related genes (IRGs) in ccRCC. Of these, hematopoietic cell signal transducer (HCST) was selected as the target gene. The HCST encodes a transmembrane signaling adaptor that forms part of the immune recognition receptor complex with the C-type lectin-like receptor NKG2D (14), which may have a role in cell survival and proliferation by activating dendritic cells (DCs), natural killer (NK) cells, and T cells (15). Thus, HCST may be a useful target for immunotherapy against ccRCC. Unfortunately, the HCST has not been studied in the field of kidney cancer.

Due to the limited understanding of the clinical significance and unique role of the HCST in ccRCC, the potential clinical value of the HCST was determined by assessment of relevant clinical data of factors and poor prognosis of ccRCC patients. Gene set enrichment analysis (GSEA) of the association between the HCST and immune cells indicated the potential role and prognostic value of the HCST in tumor immunology.

## Materials and Methods

### Human Tissue Acquisition

Human ccRCC tissues were obtained from seven male and three female patients who underwent partial nephrectomy at Zhong Nan Hospital. All samples included tumor infiltrating tissues of renal parenchyma and adjacent para-cancerous tissues, which were identified by two separate pathologists. All human samples were obtained after the approval of the Hospital Committee for Investigation in Humans and after receiving written informed consent from all patients or their relatives. All human studies were conducted in accordance with the principles of the Declaration of Helsinki.

### Data Sources

A total of 2,498 IRGs were collected from the Tumor Immune Estimation Resource (TIMER) database (https://cistrome.shinyapps.io/timer/) in May 2020 (16). The mRNA expression profiles of 539 ccRCC samples and 72 para-cancer tissue samples, as well as relevant clinical data, were downloaded from The Cancer Genome Atlas (TCGA) database (https://www.cancer.gov/about-nci/organization/ccg/research/structural-genomics/tcga) (17), of which 537 patients had matching mRNA expression profiles and survival data. In addition, two ccRCC-associated datasets (GSE53757 and GSE66272) were downloaded from the Gene Expression Omnibus (GEO) database (https://www.ncbi.nlm.nih.gov/geo/) (18). In this study, the publication guidelines of TCGA and GEO were strictly followed.

### Differential Analysis of Immune-Related Genes

The “affy” and “limma” packages in R software (https://www.r-project.org/) were used to differentiate the specimens from the GSE53757 and GES66272 datasets, which included 72 and 27 pairs of ccRCC and normal kidney specimens, respectively. Differentially expressed Immune-Related Genes (DEIRGs) were screened using *t*-test in accordance with the following cut-off values: false discovery rate (FDR) <0.05 and |log2 fold change| > 1.

### Selection of Prognostic Differentially Expressed Immune-Related Genes

Univariate (“futime” and “fustat”) Cox regression analysis (19) identified 86 DEIRGs closely correlated with the overall survival (OS) of ccRCC patients (*p* < 0.05).

### Transcription Factor Regulatory Network

Cancer associated transcription factors (TFs) were downloaded from the Cistrome Project (http://cistrome.org/), which is a comprehensive resource for predicted transcription factor targets and enhancer profiles in cancers. The correlations between TFs and the expression patterns of PDEIRGs were analyzed in order to identify the mechanism(s) underlying the dysregulation of PDEIRG expression in ccRCC. A TF regulatory network was generated using the Cytoscape_3.7.1 software (https://cytoscape.org/).

### Identification of Genes for Inclusion in a Prognostic Model

Based on the influence on the OS of ccRCC patients, the DEIRGs were screened using the Cox regression hazards model.

### Selection of the HCST Gene

Based on the data obtained from the Oncomine (https://www.oncomine.org/), Human Protein Atlas (https://www.proteinatlas.org/), and PubMed (https://pubmed.ncbi.nlm.nih.gov/) databases, the HCST gene was considered as a novel biomarker of ccRCC.

### RNA Extraction, Reverse Transcription, and Real-Time Quantitative PCR

The expression patterns of the HCST gene were assessed in matched ccRCC and para-cancerous tissues. Total RNA from tissues was isolated using the HiPurA™ Total RNA Miniprep Purification Kit (catalog no. R4111-03; Angen Biotech Co., Ltd., Guangzhou, China) in accordance with the manufacturer's instructions. The quantity of the isolated RNA was measured with a NanoDrop ND-1000 UV-Vis spectrophotometer (NanoDrop Technologies, LLC, Wilmington, DE, USA). Complementary DNA (cDNA) was synthesized from 1 μg of total RNA with the ABScript II RT Master Mix for qPCR (catalog no. RK20402; ABclonal Technology, Woburn, MA, USA). Each qPCR reaction consisted of 10 μl of 2× Universal SYBR Green Fast qPCR Mix (catalog no. RK21203; ABclonal Technology), 7 μl of ddH_2_O, 1 μl of cDNA, 1 μl of the forward primer, and 1 μl of the reverse primer. Values were normalized to that of the glyceraldehyde 3-phosphate dehydrogenase gene. A gene-specific primer pair (forward: AGG CTC TTG TTC CGG ATG TG and reverse: TAG ACT TTG CCA TCT TGG GCG) was used for amplification of the HCST gene.

### Survival Analysis

Based on the median expression value, 537 ccRCC patients were allocated to the HCST high expression group or low expression group. The R software “survival” package, Kaplan–Meier method, and log-rank test were used to evaluate the effect of the HCST on the OS of ccRCC patients. In addition, the probability (*p*) values and 95% confidence intervals (CIs) were calculated, and a survival curve was plotted (20, 21).

### Correlation Analysis of the HCST Expression Patterns and Clinicopathological Features

Clinicopathological data [i.e., age, sex, grade, TNM stage, infiltration depth (T), distant metastasis (M), and lymph node metastasis (N)] of the ccRCC tissue specimens from the TCGA database were selected for further analysis. After exclusion of incomplete or defective clinical data, data from 226 patients were included for analysis. Independent sample *t*-test and paired *t*-test were used to identify correlations between HCST expression levels and clinical-pathological parameters.

### Statistical Analysis of Potential Prognostic Factors

Potential prognostic factors were identified using the R version 4.0.2 software (“survival” and “survminer” packages). Univariate Cox regression analysis was performed to identify several prognostic factors followed by multivariate Cox regression analysis to identify independent prognostic factors.

### Protein Interaction Network Analysis

The STRING database (https://string-db.org/) (22) was used to explore the known and predicted correlations between protein interactions and HCST expression patterns, and to screen proteins that interact with the HCST.

### GSEA

The GSEA software (23) was used to divide the high and low expression groups based on the median expression value of the HCST and to detect the highest ranking gene enrichment pathways in the two groups (Molecular Signatures Database c2. Cp. Kegg. V7.2. Symbols). The Gene Matrix Transposed function dataset was used as a reference gene set for all analyses. The number of genes was set to 1,000 for the calculation of the enrichment coefficient (enrichment score) and normalized enrichment score (NES). FDR < 0.05 was considered indicative of significant enrichment.

### Correlation Analysis of HCST and Immune Cell Infiltration

The “cibersort” package (R version 4.0.2 software) was used to analyze the proportions of 22 immune cell types (LM22 gene signature) in CCRCC tissues. Then, the relationships between HCST expression levels and proportions of various immune cells were further quantified. Finally, the “ggplot2” and “limma” packages (R version 4.0.2 software) were used for analysis and plotting of the data. Meanwhile, the TIMER database was referenced for analysis of the tumor-infiltrating immune cells (i.e., CD8+ T cells, CD4+ T cells, B cells, macrophages, neutrophils, and DCs).

### Correlation Analysis of HCST and Immune-Related Genes PD-1

The expression of PD-1 is widely recognized as the most powerful predictive biomarker for anti-PD-1 therapy. The currently studied CD28 can be used as a biomarker for PD-1 expression (24). The correlations between the HCST and CD28, CD80, and CD86 were analyzed in the TIMER database to illustrate the role of the HCST as a biomarker of immunotherapy response. A correlation coefficient over 0.3 was considered statistically significant.

### Statistical Analysis

Statistical analyses were conducted using IBM SPSS Statistics for Windows, version 20.0 (IBM Corporation, Armonk, NY, USA) and R version 4.0.2. The gene expression data are presented as mean ± standard deviation. *t*-test was used to identify differences in HCST expression levels between the ccRCC and para-carcinoma tissues from the TCGA and GEO databases. Wilcoxon signed-rank test was used to analyze the interrelation between the HCST and clinical characteristic variables. Univariate and multivariate Cox analyses were used to calculate the hazard ratio and 95% CI. A *p*-value < 0.05 was considered statistically significant. FDR < 0.05 and *p* < 0.01 were considered indicative of significant enrichment.

## Results

**Figure 1:**
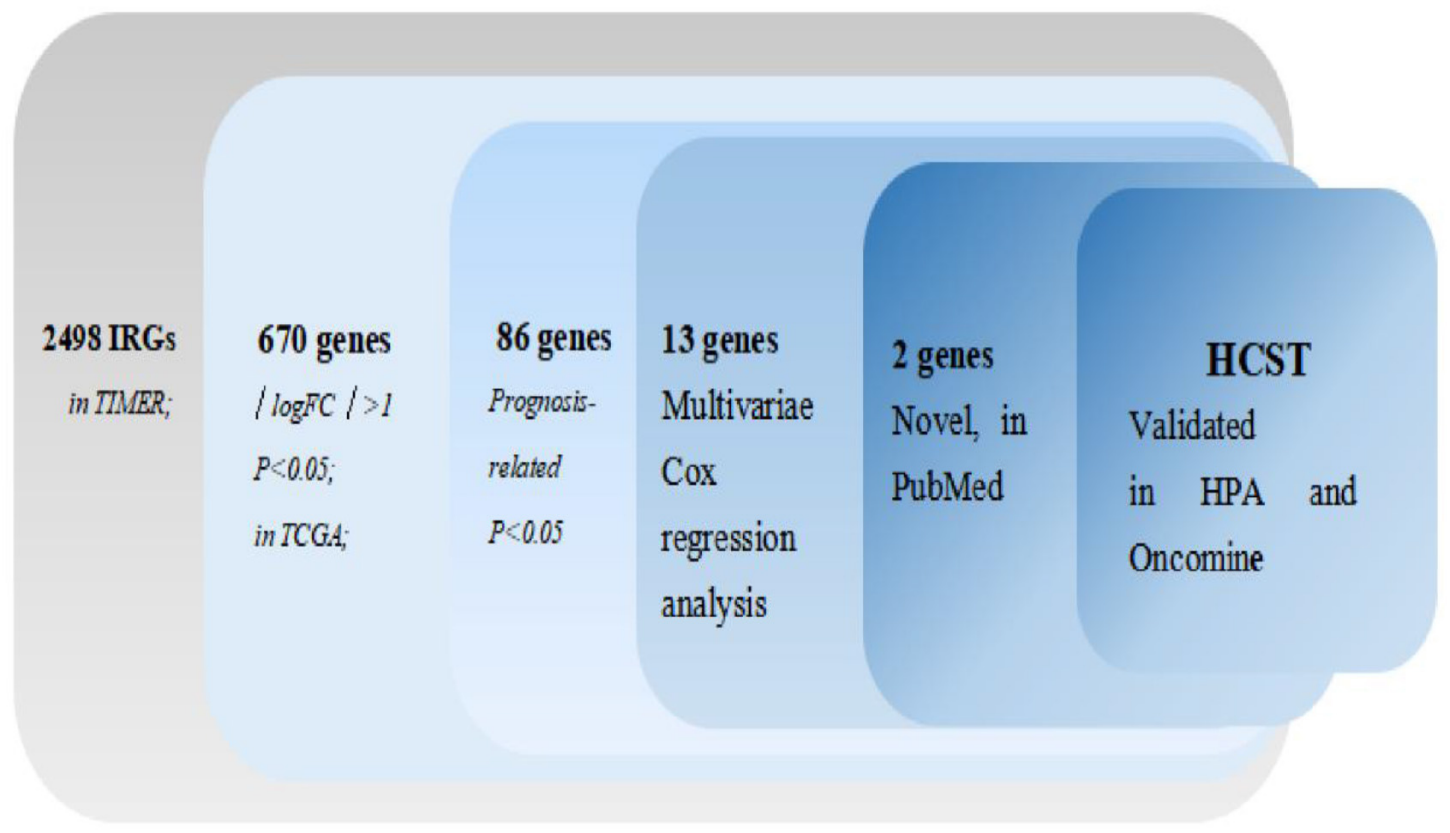
Procedure for the selection of diagnosis and prognosis biomarkers for ccRCC.

The process of screening target genes is shown in Figure 1.

### Expression Patterns of IRGs in ccRCC From Public Databases

The mRNA levels of 2,498 IRGs in 539 ccRCC samples and 72 normal renal tissue samples (TCGA) were analyzed. The same approach was applied to the GSE53757 and GES66272 datasets from the GEO database. Then, the data retrieved from two database were intersected. In total, 670 DEIRGs (554 upregulated and 116 downregulated) with an FDR < 0.05 and |log_2_ fold change| > 1 were identified.

### Identification of PDEIRGs

Univariate Cox regression analysis identified 86 PDEIRGs significantly associated with the OS and disease-free survival (DFS) of ccRCC patients (all *p* < 0.05).

### TF Regulatory Network

**Figure 2:**
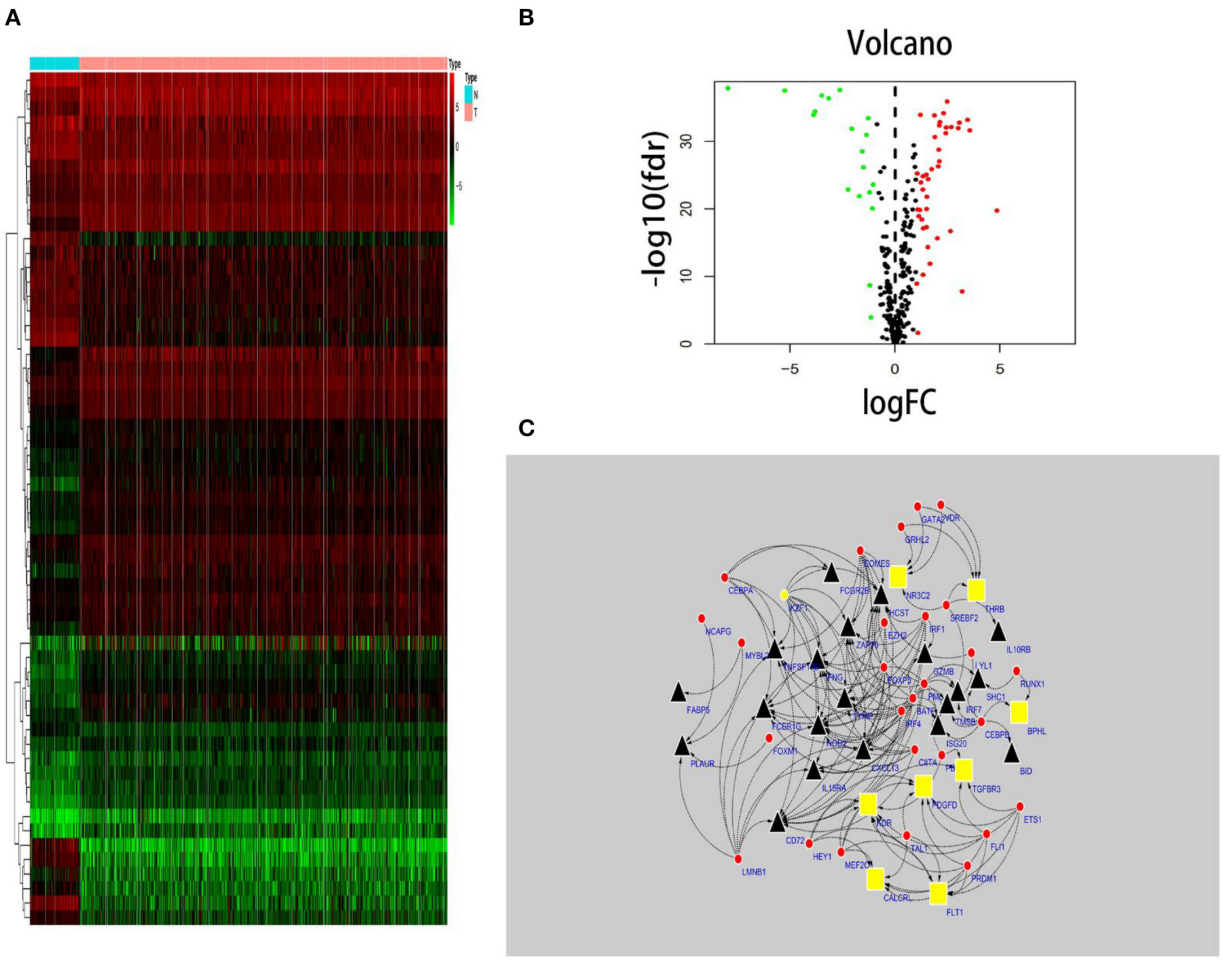
TF-based regulatory network. Construction of a TF-based regulatory network. **(A)** A heatmap of TFs differentially expressed in the tissue samples. **(B)** A volcano plot of differentially expressed TFs. **(C)** A regulatory network constructed from potentially relevant TFs (red), low-risk IRGs (red), and high-risk IRGs (black). IRGs, immune-related genes; TFs, transcription factors.

In total, 318 TFs were downloaded from the Cistrome database (http://www.cistrome.com/). Sixty TFs were significantly different at the mRNA expression levels between the ccRCC (*n* = 539) and normal renal tissue (*n* = 72) samples (*r* > 0.4 and *p* < 0.05) (Figures 2A,B). Of those 60 TFs, 28 (46.7%) turned out to be closely related to abnormal expression of PDEIRGs by using a correlation coefficient > 0.4 and a *p*-value < 0.05 as the cut-off values. Based on these data, a TF regulatory network was generated using the Cytoscape 3.7.1 software (Figure 2C).

### Establishment and Validation of an IRG-Based Prognostic Model

**Figure 3:**
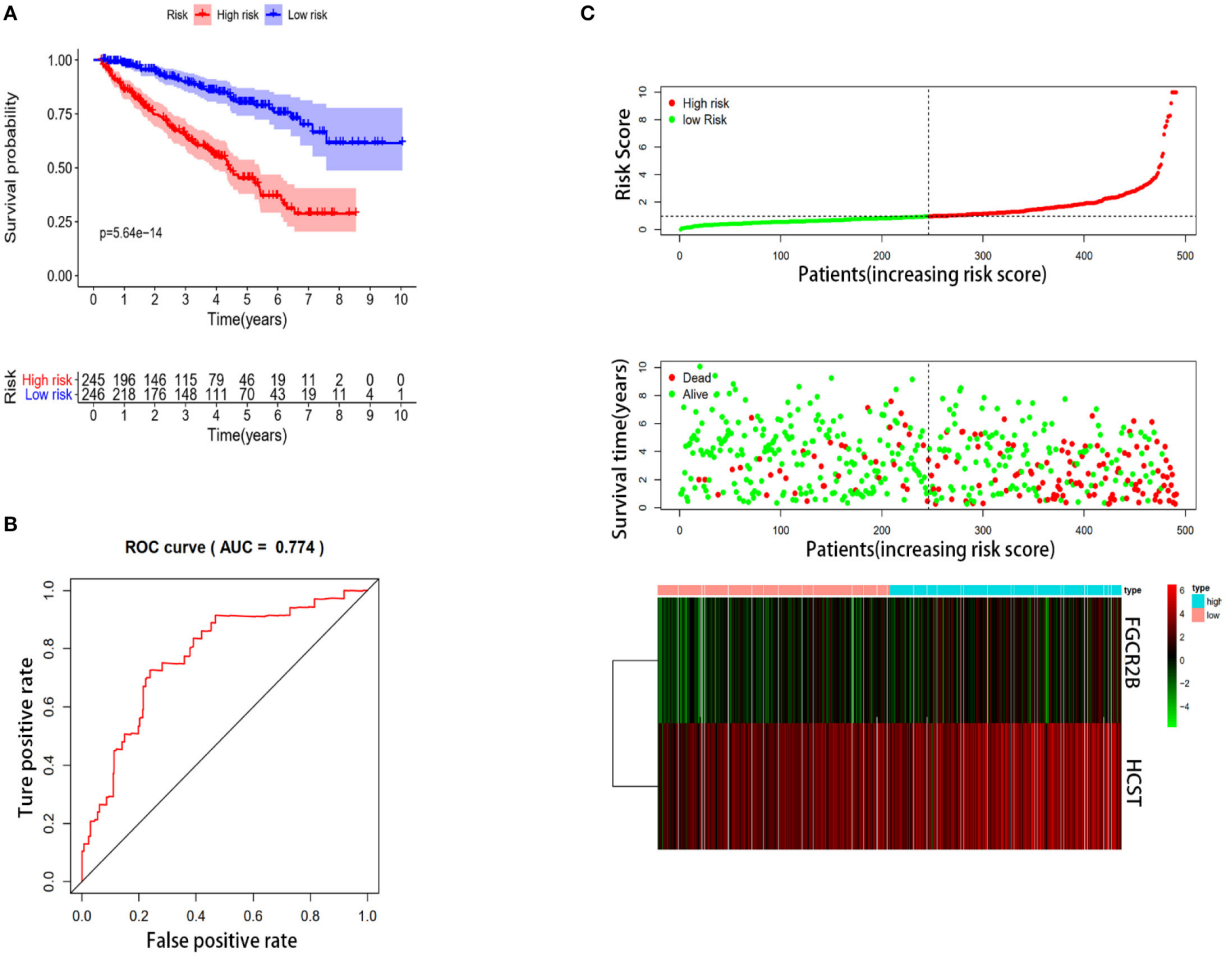
Construction of the immune-system-based risk signature by means of the training set. **(A)** Patients in the high-risk group had shorter OS. **(B)** A receiver-operating characteristic curve illustrating the prognostic value of the risk signature. **(C)** Ranking of the risk signature and distribution of the risk groups, survival status of the patients in the low-risk and high-risk groups, and a heatmap of expression profiles of the included genes.

**Figure 4:**
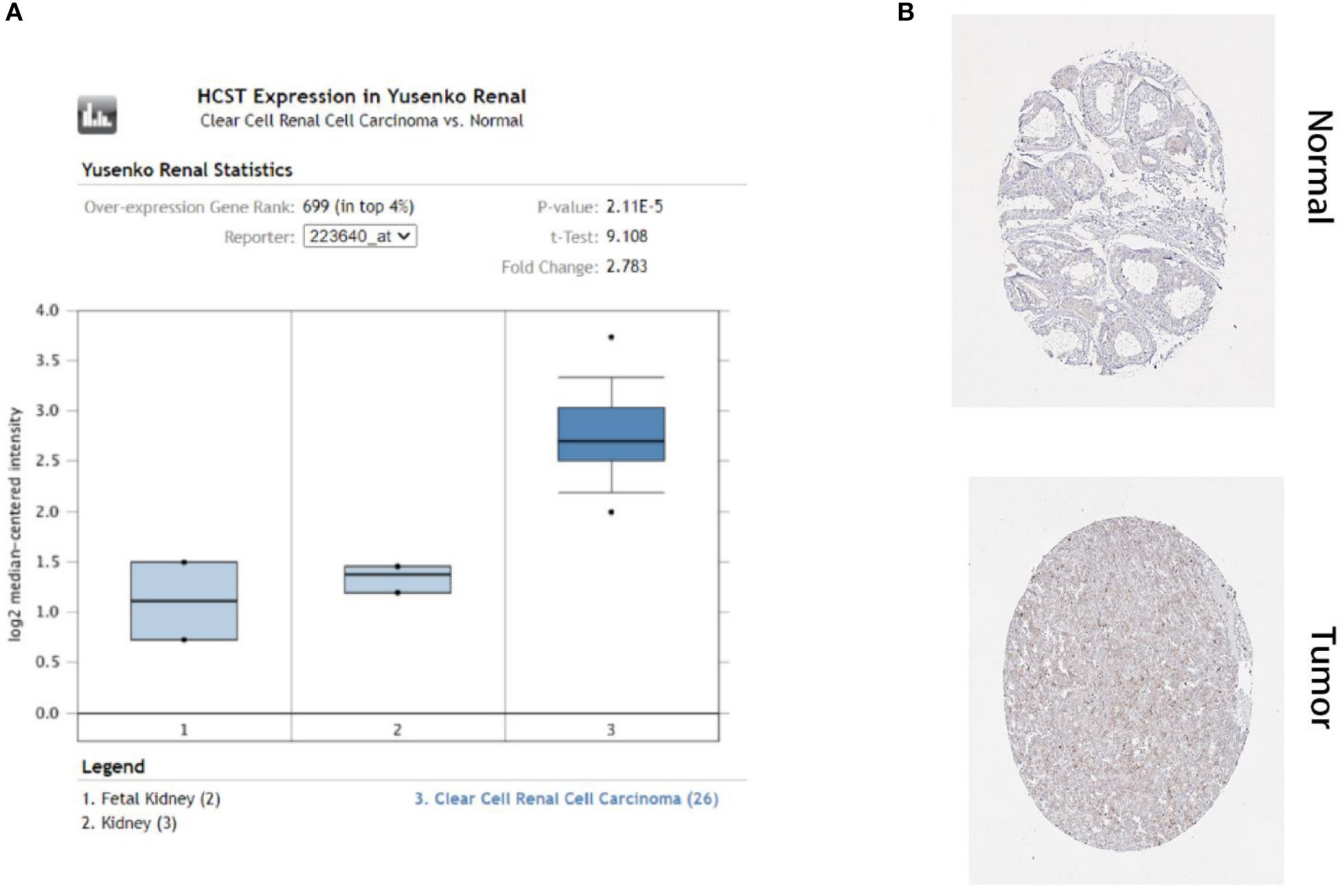
HCST protein expression was significantly higher in ccRCC tissues than normal tissues. Representative IHC images of HCST **(A)** in normal (left) and ccRCC (right) tissues. Images were downloaded from the HPA database. Statistical analyses of the protein expression levels of the HCST according to the information of normal and ccRCC tissues **(B)** from Oncomine.

In order to select the best gene model, multivariate Cox analysis was used to reduce the influence of genes on each other, and the genes with the best correlation with prognosis were selected and the risk score was calculated with the formula “Risk score (patient) $\sum_{i=1}^{N}\left(\text{expression value of }{\left(\text{gene }\right)}^{*}\text{coefficient }(\text{gene})\right)$”. In this formula, “coefficient (gene)” is the estimated regression coefficient of gene from the Cox proportional hazards regression analysis. As is shown in Supplementary Table 1, a regression risk model identified 13 PDEIRGs. To verify the accuracy and significance of the model, an OS survival curve (Figure 3A), a receiver-operating characteristic curve (Figure 3B), and a risk curve of the IRG-based prognosis model (Figure 3C) were generated. A search of the PubMed database (performed on 2 May, 2020) revealed 11 genes associated with ccRCC in the model, which did not include the HCST and FCGR2. According to the Beroukhim dataset derived from the Oncomine database, the fold change of these two genes was >2. But only HCST overexpression was ranked in the top 5% (Figure 4A). Analysis of 36 histological section images of ccRCC and normal kidney tissues from the HPA database showed that HCST protein expression was significantly increased in ccRCC tissues (Figure 4B). Therefore, the HCST was chosen for further analysis.

### Experimental Validation

**Figure 5:**
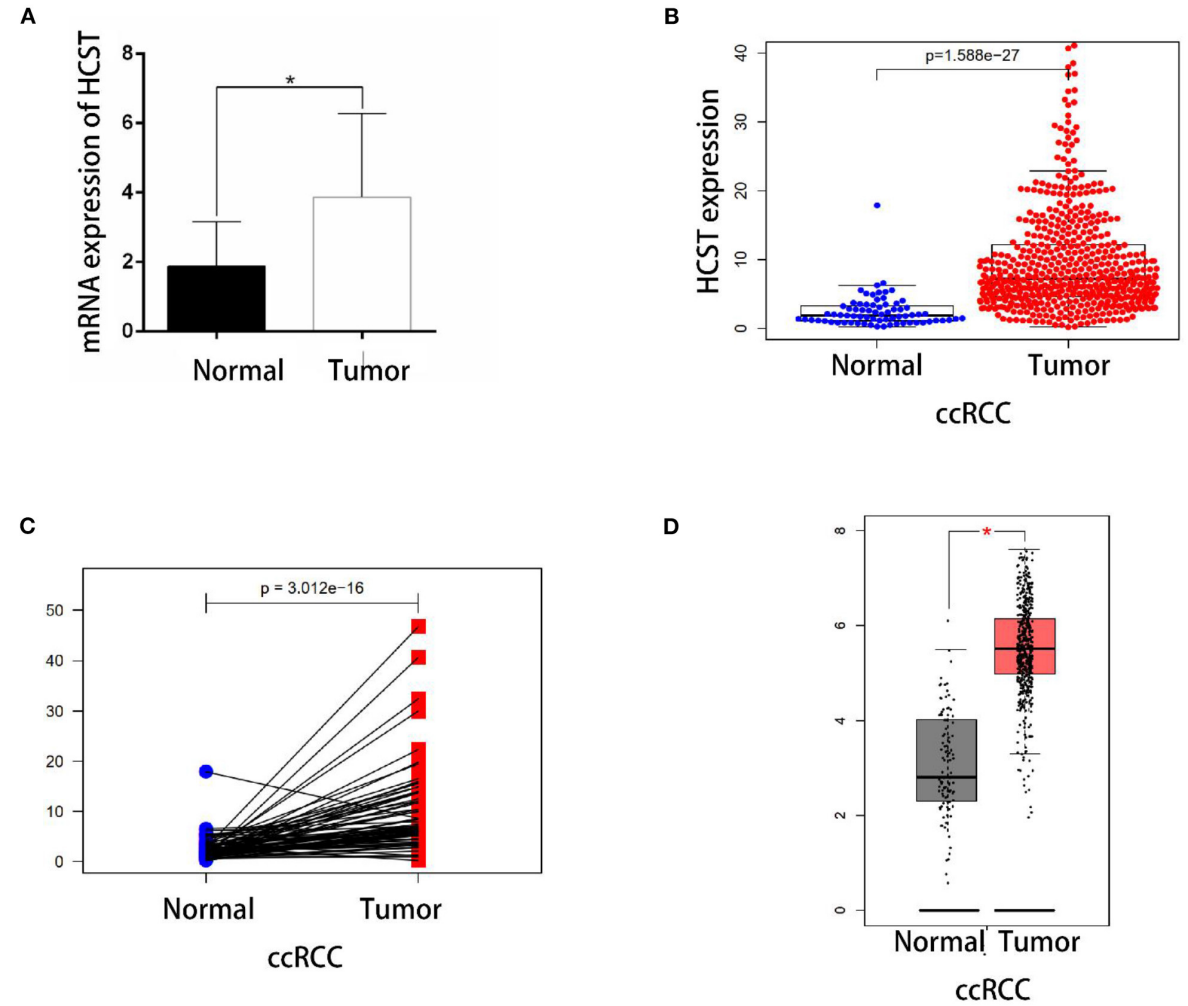
Experimental validation. **(A)** Detection of HCST mRNA expression levels in 10 cases of renal carcinoma and para-cancer tissue by qRT-PCR. The glyceraldehyde 3-phosphate dehydrogenase gene was used as an internal control. HCST expression in cancer cells is clearly higher than normal kidney cells. **(B)** Statistical analyses of the mRNA expression levels of the HCST according to the information of normal and ccRCC tissues from R version 4.0.2 software matching TCGA data. **(C)** Statistical analyses of the mRNA expression levels of the HCST according to cancer and para-cancerous tissue from the same ccRCC patient from R version 4.0.2 software matching TCGA data. **(D)** Statistical analyses of the mRNA expression levels of the HCST according to the information of normal and ccRCC tissues from GEPIA 2 matching TCGA and GTEx data. *p < 0.05.

qRT-PCR analysis showed that HCST mRNA levels were significantly higher in ccRCC tissues than those in normal renal tissues (Figure 5A). Consistently, the HCST was observed upregulated with the R version 4.0.2 software analysis of TCGA data (Figures 5B,C), of which HCST mRNA levels of cancer and para-cancerous tissue are from the same ccRCC patients (Figure 5C). Matching TCGA and GTEx data, the Gene Expression Profiling Interactive Analysis (GEPIA2) (http://gepia.cancer-pku.cn/) found similarly elevated HCST expression (Figure 5D).

### Relationship Between HCST Gene Expression Levels and Clinicopathological Indices of Tumor Tissues

A median gene expression value of 6.436 was used to stratify the 537 TCGA-ccRCC patients into the low or high expression group. Analysis using TCGA clinical data and R version 4.0.2 showed that HCST expression was correlated with grade (*p* = 0.005), TNM stage (*p* = 0.001), lymph node metastasis (*p* = 0.004), and invasion depth (*p* = 0.018), but not age (*p* = 0.721), sex (*p* = 0.292), or distant metastasis (*p* = 0.218) (Table 1).

**Table 1:** Relationship between HCST expression level and clinicopathological variables in ccRCC patients.

**Classification**	**Total**	**HCST expression**	***t***	***P***
**Age**				
≤ 60	142	10.530 ± 10.300	0.709	0.721
>60	84	11.077 ± 11.169		
**Gender**				
Male	141	10.805 ± 12.080	0.897	0.292
Female	85	10.615 ± 7.637		
**TMN stage**				
I–II	122	9.068 ± 9.300	0.010	0.001
III–IV	104	12.630 ± 11.712		
**Grade**				
G1–G2	98	9.013 ± 9.765	0.031	0.005
G3–G4	128	12.075 ± 11.078		
**Invasion depth**				
T1–T2	134	9.798 ± 9.897	0.110	0.018
T3–T4	92	12.097 ± 11.489		
**Lymph node metastasis**				
N0	213	10.126 ± 9.167	<0.001	0.004
N1	13	20.690 ± 22.631		
**Distant metastasis**				
M0	186	10.479 ± 10.920	0.431	0.128
M1	38	11.953 ± 9.005		

*p < 0.05, statistically significant*.

### HCST Is an Independent Poor Prognostic Factor of ccRCC

**Figure 6:**
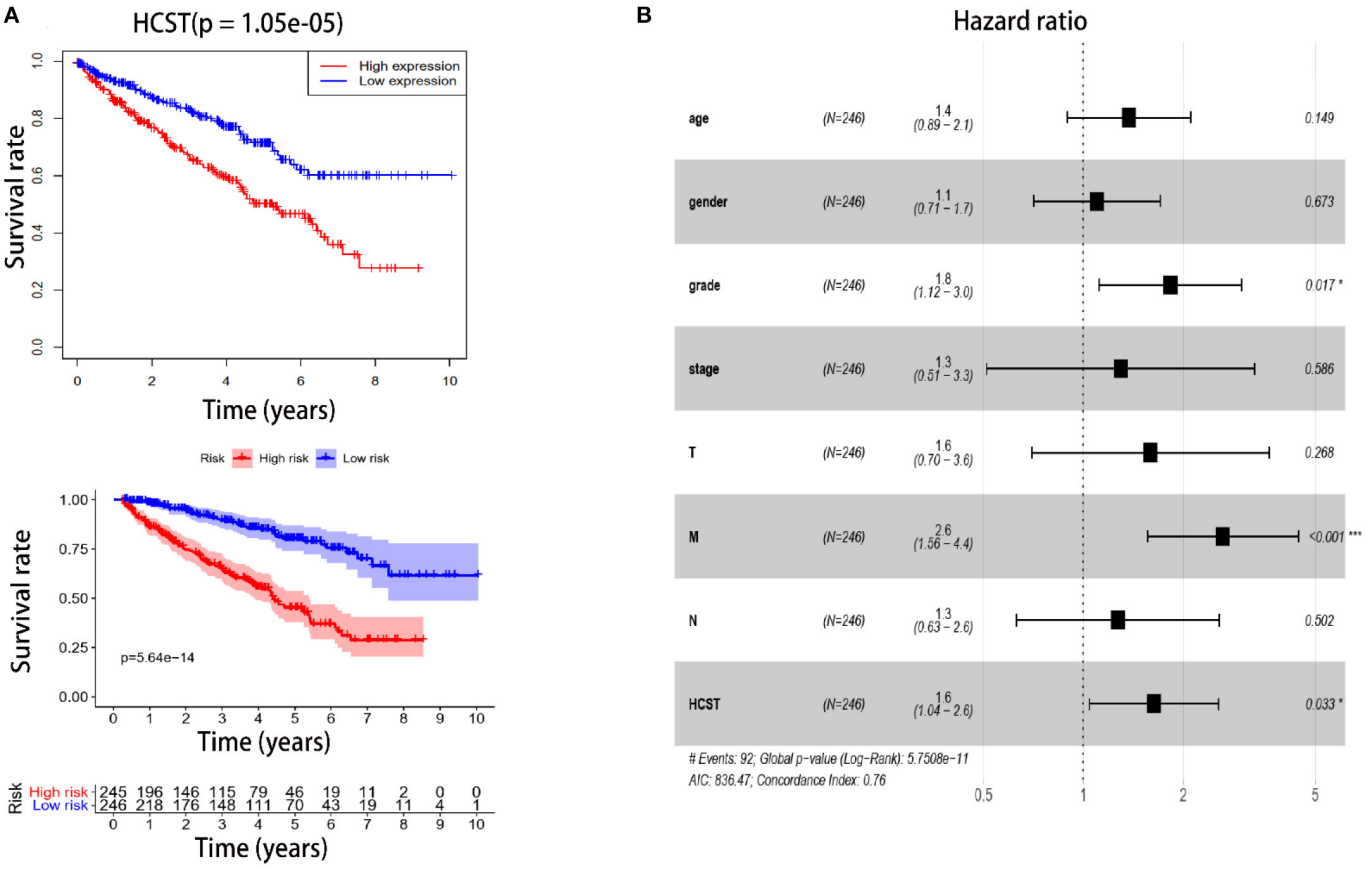
High HCST expression is associated with poor survival of ccRCC patients. **(A**, Upper**)** OS of HCST (high) and HCST (low) ccRCC patients matching the TCGA database from R version 4.0.2 software. **(A**, Down**)** DFS of HCST (high) and HCST (low) patients from the GEPIA2 matching TCGA and GTEx data. (**B)** Multivariate Cox analysis showing the hazard ratios of different factors. The number of events for the number of tested factors was 92. The global *p*-value (log-rank) was 5.7508e−05, Akaike's information criterion was 836.47, and the concordance index was 0.76.

The R software “survival” package, Kaplan–Meier method, and log-rank test were used to assess the effect of the HCST on the OS of ccRCC patients. The logarithmic rank *p*-value and 95% CI were calculated. Then, a survival curve was plotted. Univariate and multivariate Cox regression analyses were performed to investigate whether high expression of the HCST could be an independent adverse prognostic factor in patients with ccRCC. As shown in Table 2, Cox univariate survival analysis indicated that grade (*p* < 0.001), TNM stage (*p* < 0.001), lymph node metastasis (*p* = 0.001), invasion depth (*p* < 0.001), distant metastasis (*p* < 0.001), and HCST expression (*p* = 0.005) were important parameters affecting the duration of OS, while multivariate Cox survival analysis showed that grade, distant metastasis, and HCST expression were independent factors of a poor prognosis of ccRCC patients (all, *p* < 0.05) (Figure 6).

**Table 2:** Univariate analysis of the prognostic factors in ccRCC patients using a Cox regression model.

**Parameters OS**	**Univariate analysis**		**Multivariate analysis**	
	***HR(95%CI)***	***p***	***HR*(*95%CI)***	***p***
HCST expression High vs. Low	1.853(1.210–2.839)	0.004	1.630 (1.042–2.552)	0.032
Age ≥65 vs. <60	1.370(0.908–2.067)	0.133	1.371 (0.893–2.105)	0.149
Female vs. male	1.013(0.666–1.541)	0.951	1.099 (0.709–1.704)	0.673
TMN stage III/IV vs. I/II	3.676(2.366–5.711)	<0.001	1.295 (0.511–3.280)	0.278
Grade G1/2 vs. G3/4	2.629(1.655–4.176)	<0.001	1.829 (1.115–3.001)	0.017
Invasion depth T1/2 vs. T3/4	3.311(2.167–5.058)	<0.001	1.594 (0.699–3.634)	0.268
Lymph node metastasis	2.932(1.516–2.839)	<0.001	1.273 (0.629–2.574)	0.502
Distant metastasis	4.073(2.634–6.300)	0.001	2.634 (1.562–4.442)	<0.001

*CI, confidence interval*.

### Protein Interaction Network of HCST

**Figure 7:**
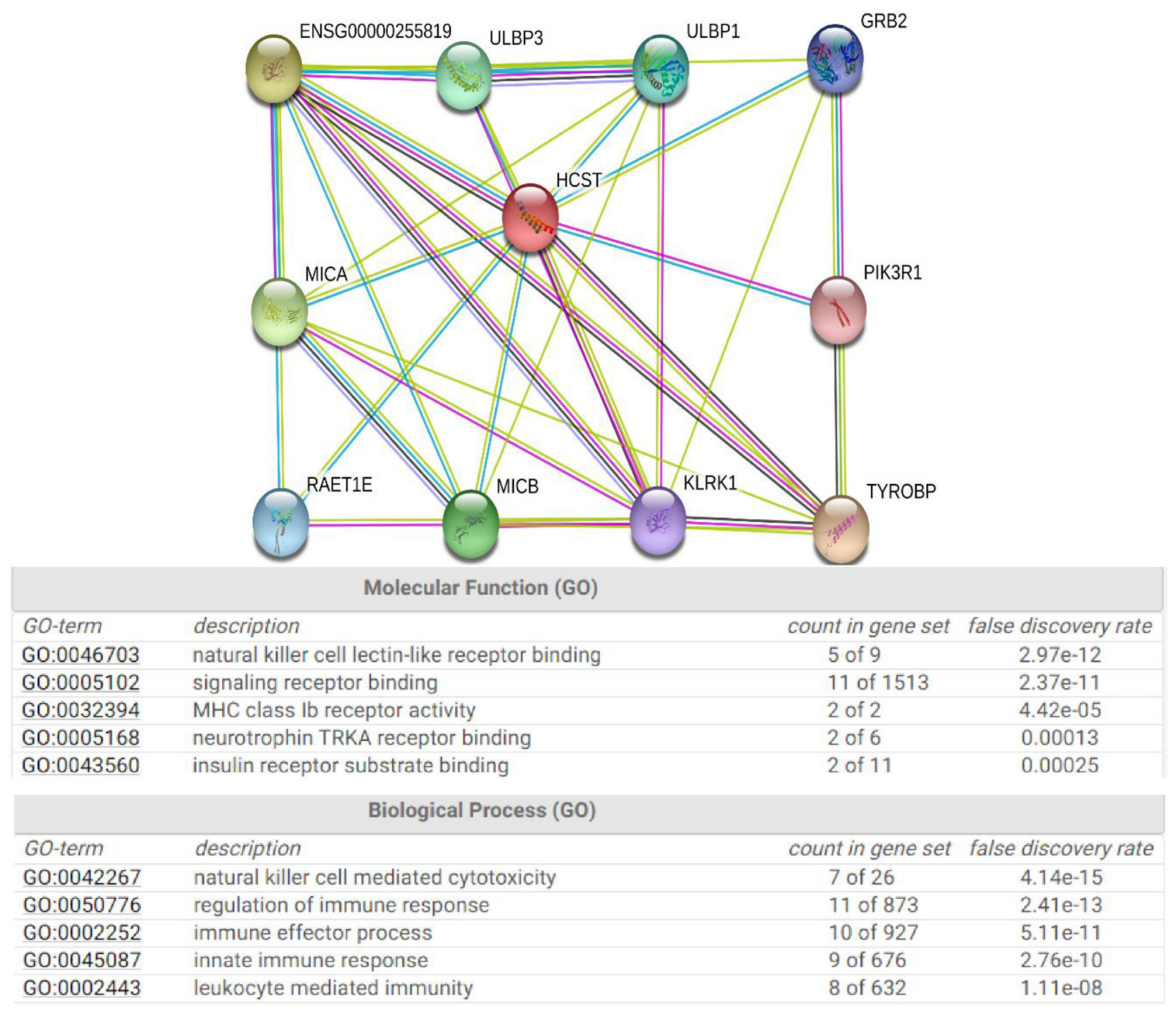
Protein interaction network of HCST. An interaction network of the HCST protein with other proteins (i.e., TYROBP, KLRC4, MICA, MICB, ULBP3, ULBP1, RAET1E, GRB2, KLRK1, and PIK3R1). The interaction network was obtained from the STRING database.

The STRING database was used to explore the known and predicted protein–protein associations involving HCST. The top 10 predicted functional partners were TYROBP (score = 0.983), KLRC4 (score = 0.976), MICA (score = 0.966), MICB (score = 0.965), ULBP3 (score = 0.962), ULBP1 (score = 0.962), RAET1E (score = 0.951), GRB2 (score = 0.942), KLRK1 (score = 0.923), and PIK3R1 (score = 0.870) (Figure 7). Function enrichment analysis of the HCST gene revealed that the most significant biological processes were “natural killer cell-mediated cytotoxicity,” “regulation of immune response,” “immune effector process,” and “innate immune response.” In regard to cellular components, the HCST gene was significantly enriched in “cell surface,” “the plasma membrane,” “membrane part,” and “intrinsic component of plasma membrane.”

### GSEA of HCST

**Figure 8:**
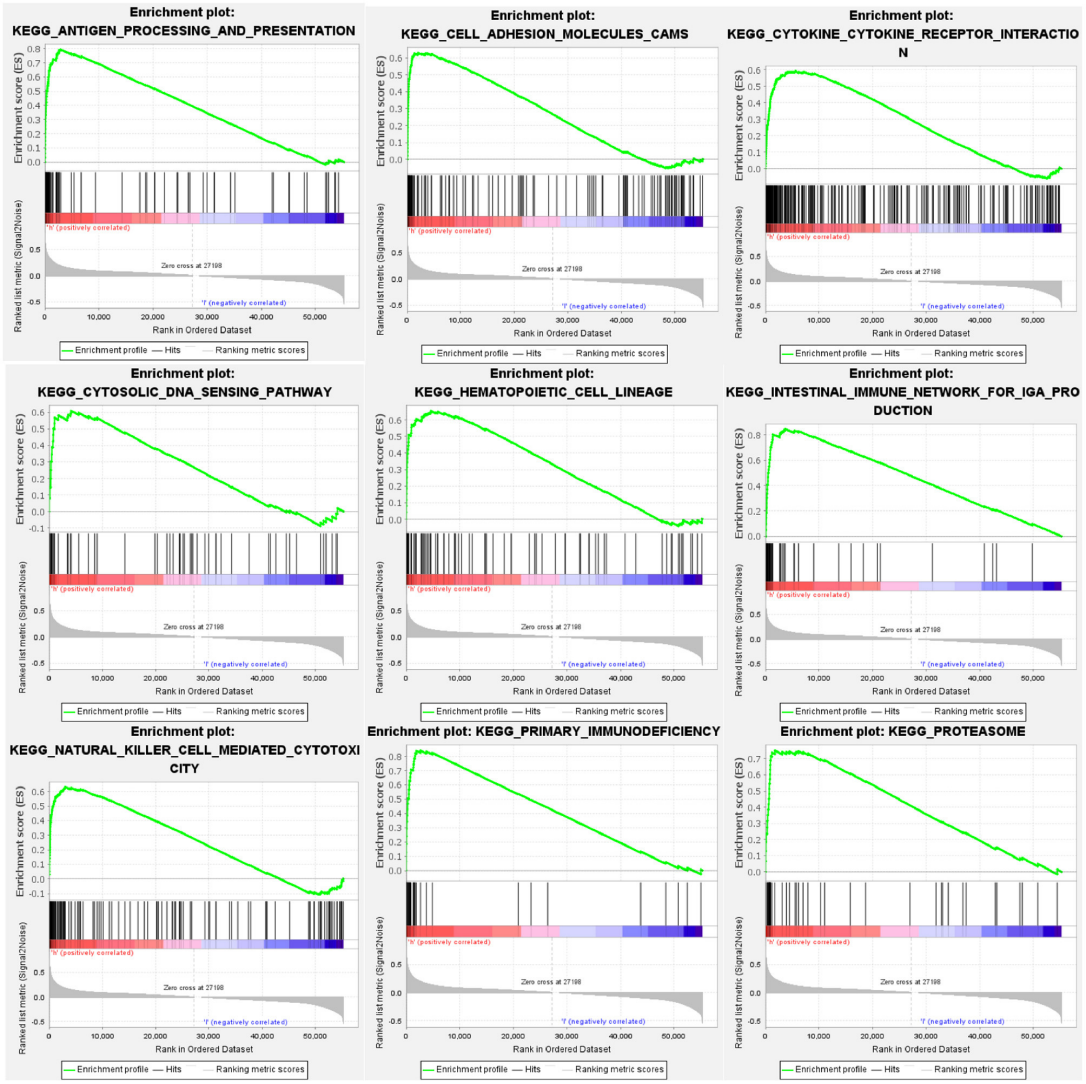
GSEA identification of HCST-related signaling pathways in ccRCC. GSEA pathways enriched in samples with high HCST expression. The GSEA results showed that the terms “proteasome,” “cytosolic DNA sensing pathway,” “cell adhesion molecules cams,” “cytokine receptor interaction,” “primary immunodeficiency,” “hematopoietic cell lineage,” “natural killer cell-mediated cytotoxicity,” “intestinal immune network for IGA production,” and “antigen processing and presentation” were differentially enriched in GC samples with high BICC1. NES, normalized enrichment score.

GSEA identified 57 HCST-related signaling pathways that were upregulated in ccRCC, 17 of which were more obviously enriched (NOM *p* < 0.05, FDR <0.1, and NES > 2.0) (Figure 8). As shown in Table 3, the terms “proteasome,” “cytosolic DNA sensing pathway,” “cell adhesion molecules cams,” and “cytokine receptor interaction,” whose function was involved in cell adhesion and tumorigenesis, were significantly enriched in the HCST high expression group. Meanwhile, the terms associated with immune and inflammatory responses included “hematopoietic cell lineage,” “intestinal immune network for IGA production,” “natural killer cell-mediated cytotoxicity,” “antigen processing and presentation,” and “primary immunodeficiency.”

**Table 3:** GSEA pathways upregulated due to high expression of HCST.

**GS <br> follow link to MSigDB**	***ES***	***NES***	***p***	***FDR***
KEGG_PROTEASOME	0.75	2.09	0.004	0.004
KEGG_CYTOSOLIC_DNA_SENSING_PATHWAY	0.61	2.21	<0.001	0.001
KEGG_CELL_ADHESION_MOLECULES_CAMS	0.63	2.30	<0.001	<0.001
KEGG_CYTOKINE_CYTOKINE_RECEPTOR_INTERACTION	0.59	2.47	<0.001	<0.001
KEGG_PRIMARY_IMMUNODEFICIENCY	0.84	2.20	<0.001	0.001
KEGG_NATURAL_KILLER_CELL_MEDIATED_CYTOTOXICITY	0.63	2.38	<0.001	<0.001
KEGG_INTESTINAL_IMMUNE_NETWORK_FOR_IGA_PRODUCTION	0.85	2.51	<0.001	<0.001
KEGG_ANTIGEN_PROCESSING_AND_PRESENTATION	0.79	2.60	<0.001	<0.001
KEGG_HEMATOPOIETIC_CELL_LINEAGE	0.65	2.26	<0.001	0.001

*NES, normalized enrichment score; NOM, nominal; FDR, false discovery rate. Gene sets with NOM p-value < 0.05 and FDR q-value < 0.1 are considered as significant*.

### Interrelation With Tumor-Infiltrating Immune Cells in ccRCC

**Figure 9:**
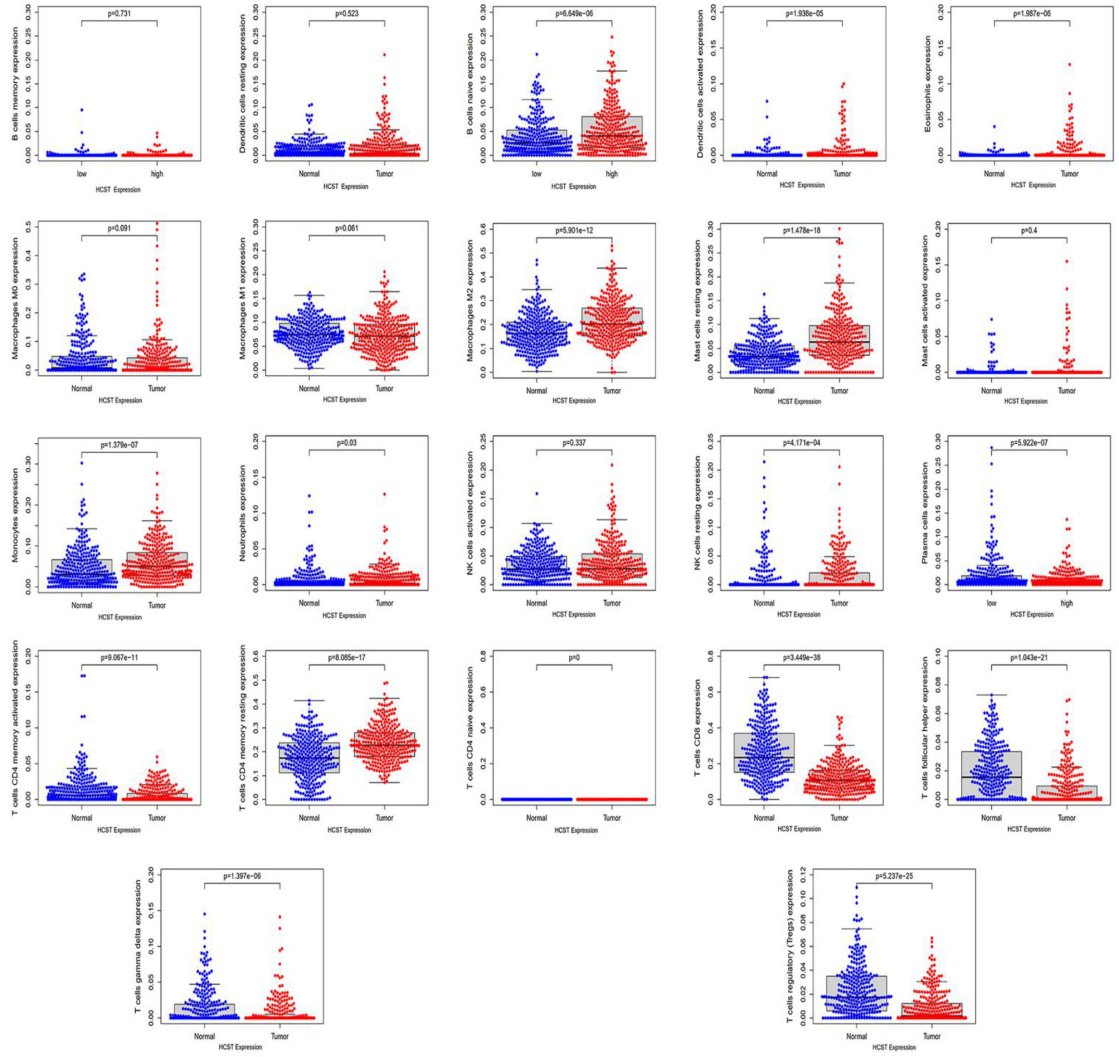
HCST was significantly correlated with tumor-infiltrating immune cells in ccRCC. Analysis of the TCGA dataset via the LM22 signature matrix using CIBERSORT online. In total, 22 kinds of tumor-infiltrating immune cells are plotted according to the HCST expression level. There were significant differences in naïve B cells, activated DCs, eosinophils, M2 macrophages, resting mast cells, monocytes, neutrophils, resting NK cells, plasma cells, activated CD4 memory T cells, resting CD4 memory T cells, CD8 T cells, follicular helper T cells, gamma delta T cells, and regulatory T cells (all, *p* < 0.001).

**Figure 10:**
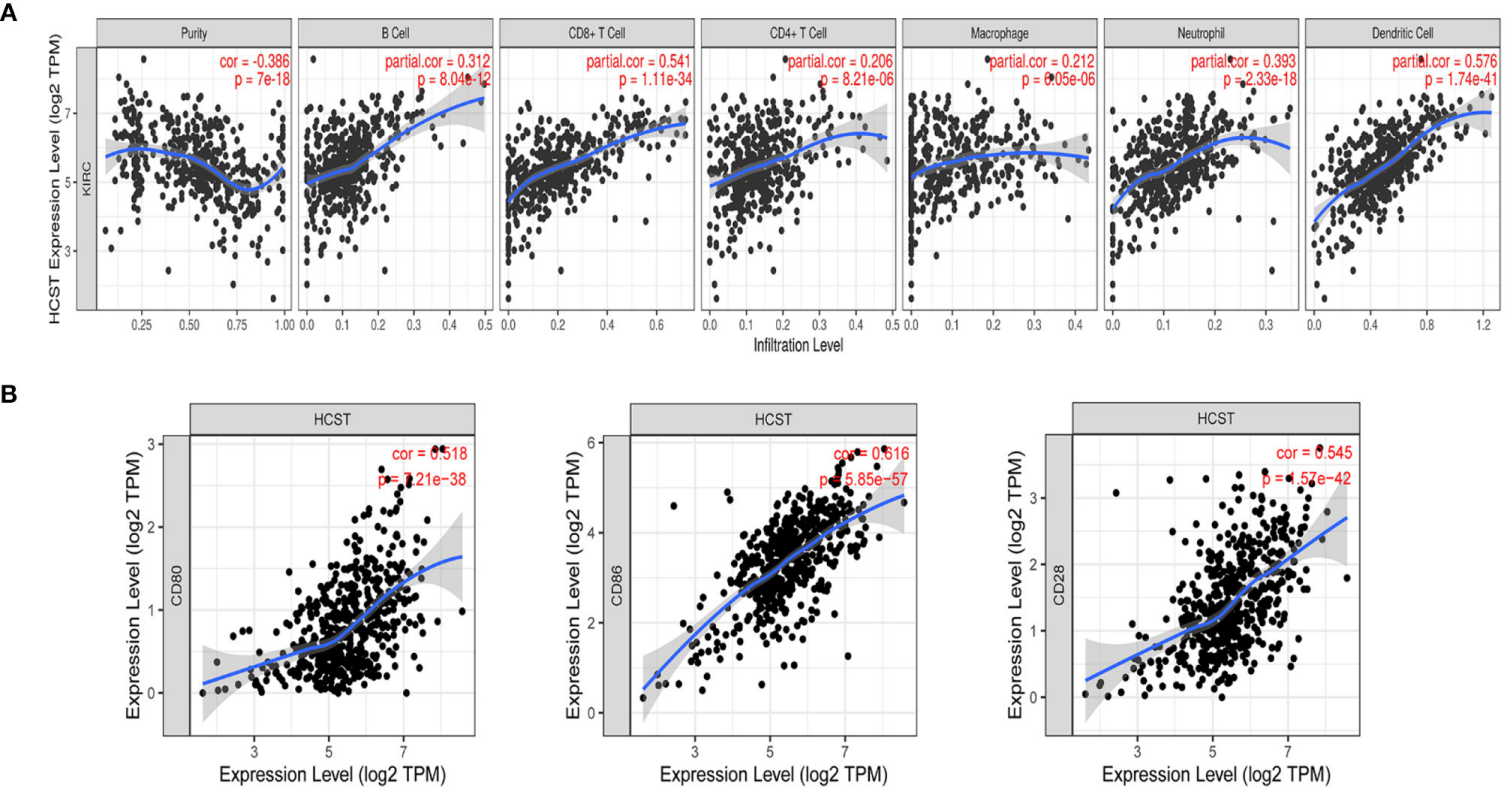
Correlation of HCST with tumor-infiltrating immune cells in ccRCC and the expression of the immune-related genes PD-1. **(A)** In TIMER, HCST expression was correlated with B cells (*r* = 0.312, *p* = 8.04e−12), CD8+ T cells, **(A)** (*r* = 0.541, *p* = 1.11e−34) and neutrophils (*r* = 0.3.93, *p* =2.33e−18), and strongly correlated with DCs (*r* = 0.576, *p* = 1.74e−41). **(B)** HCST with CD80, CD86, and CD28 in ccRCC.

Analysis with the CIBERSOFT software showed that HCST expression was correlated with tumor-filtrating immune cells, including naïve B cells, activated DCs, eosinophils, M2 macrophages, resting mast cells, monocytes, neutrophils, resting NK cells, plasma cells, activated CD4 memory T cells, resting CD4 memory T cells, CD8 T cells, follicular helper T cells, gamma delta T cells, and regulatory T cells (all, *p* < 0.001) (Figure 9). In addition, the TIMER database indicated that HCST expression was positively correlated to the levels of different infiltrating immune cells, including B cells (*r* = 0.312, *p* = 8.04e−12), CD8+ T cells (*r* = 0.541, *p* = 1.11e−34), and neutrophils (*r* = 0.3.93, *p* =2.33e−18), and strongly correlated with DCs (*r* = 0.576, *p* = 1.74e−41) (Figure 10A).

### The Correlation Analysis of HCST and Immune-Related Genes PD-1

Our results showed that the HCST was positively correlated with the expression of CD80 in ccRCC (cor = 0.518, *p* = 7.21e-38, respectively); the HCST was positively correlated with the expression of CD86 in ccRCC (cor = 0.545, *p* = 1.57e-42, respectively); the HCST was positively correlated with the expression of CD28 in ccRCC (cor = 0.616, *p* = 5.85e-57, respectively) (Figure 10B).

## Discussion

In recent years, due to the continuous and stable antitumor responses, immunotherapy has become the first-line therapy for ccRCC. Various studies of immunotherapy regimens have revealed that immune cell infiltration and IRGs play pivotal roles in carcinogenesis and tumor progression (25, 26). However, the relationship between IRGs and the mechanisms underlying tumorigenesis and progression is still not fully understood in ccRCC.

In the present study, IRG expression levels in ccRCC tissues were analyzed systematically. With a multistep selection and validation procedure, the HCST gene was chosen as the proposed IRG-based prognostic model. Firstly, R version 4.0.2 software was used to analyze the transcriptomic and clinical data retrieved from TCGA, which showed that patients had significantly shorter durations of OS and DFS with higher HCST mRNA levels. In addition, high HCST expression has been associated with grade (*p* = 0.005), TNM stage (*p* = 0.001), lymph node metastasis (*p* = 0.004), and invasion depth (*p* = 0.018) in ccRCC. Moreover, univariate and multivariate analyses demonstrated that the HCST was an independent poor prognostic biomarker of OS and DFS in ccRCC patients.

Subsequently, GSEA was performed with the STRING database to determine the molecular functions and potential mechanisms of the HCST. Protein–protein interaction analysis showed that the top 10 proteins associated with the HCST included TYROBP, KLRC4, MICA, MICB, ULBP3, ULBP1, RAET1E, GRB2, KLRK1, and PIK3R1, which are mainly involved in the immune response and tumorigenesis. Functional enrichment analysis of these interaction partners at the gene level showed enrichment in the terms “immunoreaction” and “encoding a transmembrane signaling adaptor.” For instance, PIK3R1 is a major regulatory isomer of PI3K, and dysregulation of the PI3K/PTEN pathway is a common cause of cancer (27). The HCST may be involved in tumorigenesis through synergistic action with these genes. The GSEA study further indicated that the pathways enriched in tissue samples with high HCST expression were mainly related to cell adhesion, tumor formation, and the immune response. Of nine representative upregulated pathways, the enriched terms “proteasome,” “cytosolic DNA sensing pathway,” “cell adhesion molecules cams,” and “cytokine receptor interaction” were associated with cell adhesion and tumorigenesis, while “hematopoietic cell lineage,” “intestinal immune network for IGA production,” “natural killer cell-mediated cytotoxicity,” “antigen processing and presentation,” and “primary immunodeficiency” were correlated to immune and inflammatory responses. Hence, these findings uncovered the molecular functions and underlying mechanisms of the HCST in ccRCC. High expression of the HCST influences the occurrence and development of ccRCC and contributes to the unfavorable prognosis of ccRCC patients.

Based on differential HCST expression, CIBERSORT analysis was used to evaluate the estimated proportions of tumor-infiltrating immune cells in ccRCC, which included naïve B cells, activated DCs, eosinophils, M2 macrophages, resting mast cells, monocytes, neutrophils, resting NK cells, plasma cells, activated CD4 memory T cells, resting CD4 memory T cells, CD8 T cells, follicular helper T cells, gamma delta T cells, and regulatory T cells. The expression level of the HCST influenced the proportions of these immune cells. Further analysis with the use of the TIMER database revealed that the HCST gene was prominently correlated with the tumor infiltration of B cells, CD8+ T cells, and neutrophils and strongly interrelated with DCs. Barry et al. found that intratumorally stimulatory DCs play important roles in the stimulation of cytotoxic T cells and driving the immune responses against cancer (28). Additionally, DCs were found to play a central role in the regulation of the balance between CD8 T-cell immunity vs. tolerance to tumor antigens (29–31). Of the antigen-presenting cells, DCs are the most effective in the activation of naïve T cells and induce an immune memory response in cancer (32). A number of effective tumor treatments related to DCs have been proposed, such as administration in conjunction with (neo)antigens, mobilization of endogenous DCs, and the use of stimulating adjuvants (33). However, improvements to treatment strategies are still required to identify and understand biomarkers associated with DCs. Our study suggested that the HCST could influence the prognosis of ccRCC by affecting tumor-related immune cells, especially DCs.

Notably, T cell activation is dependent upon signals delivered through the antigen-specific T cell receptor and accessory receptors on the T cell. PD-1 is an inhibitory receptor with two B7-like ligands. A primary costimulatory signal is delivered through the CD28 receptor with combining its ligands, B7-1 (CD80) or B7-2 (CD86) (34). Therefore, CD28 can be used as a responsive biomarker to the expression of the IRGs PD-1. Therefore, the expression of the HCST can play roles in predicting the response to anti-PD-1 therapy in ccRCC.

Finally, we discovered, for the first time, the effect of the HCST on ccRCC. Consistently, Milioli et al. found that high HCST expression was associated with poor survival of patients with basal-like breast cancer, the cancer immune response, epithelial-mesenchymal transition, and the cell cycle (35). Qi et al. found that the HCST might be potential novel predictive markers for immunotherapy in non-small cell lung cancer (24). We performed a primary test using qRT-PCR to determine the expression of the HCST in renal cancer tissues and compared them with para-cancer tissues. Moreover, we conducted a survival analysis to verify the prognostic value of the HCST by extracting data from the TCGA database. However, a second cohort study will be more convincing if validated. Additionally, it is worth performing experimental studies on specific mechanisms. Therefore, further investigations are required.

In summary, the present study verified that overexpression of the HCST was interrelated to the clinicopathology and poor prognosis of ccRCC. High HCST expression was also closely correlated with the levels of tumor-infiltrating immune cells, especially DCs. However, further studies of the molecular function of the HCST are needed to identify new targets for immunotherapy of ccRCC, as well as new biomarkers for prognostic prediction.

## Data Availability Statement

Publicly available datasets were analyzed in this study. This data can be found here: https://portal.gdc.cancer.gov/repository?facetTab=files&filters=%7B%22op%22%3A%22and%22%2C%22content%22%3A%5B%7B%22op%22%3A%22in%22%2C%22content%22%3A%7B%22field%22%3A%22cases.primary_site%22%2C%22value%22%3A%5B%22kidney%22%5D%7D%7D%2C%7B%22op%22%3A%22in%22%2C%22content%22%3A%7B%22field%22%3A%22cases.project.program.name%22%2C%22value%22%3A%5B%22TCGA%22%5D%7D%7D%2C%7B%22op%22%3A%22in%22%2C%22content%22%3A%7B%22field%22%3A%22cases.project.project_id%22%2C%22value%22%3A%5B%22TCGA-KIRC%22%5D%7D%7D%2C%7B%22op%22%3A%22in%22%2C%22content%22%3A%7B%22field%22%3A%22files.analysis.workflow_type%22%2C%22value%22%3A%5B%22HTSeq%20-%20FPKM%22%5D%7D%7D%2C%7B%22op%22%3A%22in%22%2C%22content%22%3A%7B%22field%22%3A%22files.data_type%22%2C%22value%22%3A%5B%22Gene%20Expression%20Quantification%22%5D%7D%7D%2C%7B%22op%22%3A%22in%22%2C%22content%22%3A%7B%22field%22%3A%22files.experimental_strategy%22%2C%22value%22%3A%5B%22RNA-Seq%22%5D%7D%7D%5D%7D https://www.ncbi.nlm.nih.gov/geo/query/acc.cgi?acc=GSE53757 https://www.ncbi.nlm.nih.gov/geo/query/acc.cgi https://cistrome.shinyapps.io/timer/ http://cistrome.org/CistromeCancer/CancerTarget/.

## Author Contributions

YZ, DL, XW, and HL designed the experiment. YZ wrote the first draft of the manuscript. YZ, XW, WZ, HL, and DL conducted most of the experiments and performed the analysis procedures. PC, DX, JL, GZ, ML, ZW, XW, and YGZ helped to analyze the results. MD and XZ critically revised drafts of the manuscript, provided important intellectual input, and approved the final version for publication. YZ and XZ contributed to the writing of the manuscript. All authors contributed to the article and approved the submitted version.

## Conflict of Interest

The authors declare that the research was conducted in the absence of any commercial or financial relationships that could be construed as a potential conflict of interest.

## Abbreviations

ccRCC: Clear cell renal cell carcinoma
TCGA: The Cancer Genome Atlas
GEO: Gene Expression Omnibus
IRG: Immune-related gene
HCST: Hematopoietic cell signal transducer
GSEA: Gene set enrichment analysis
OS: Overall survival
CTLA4: Cytotoxic T lymphocyte-associated antigen 4
PD-1: Programmed cell death protein 1
PD-L1: Programmed death-2
DCs: Dendritic cells
NK cells: Natural Killer cells
TIMER: Tumor Immune Estimation Resource
TF: Transcription factors.
