# Supplementary material for: The Immune-Related Gene HCST as a Novel Biomarker for the Diagnosis and Prognosis of Clear Cell Renal Cell Carcinoma
Source: Front Oncol. 2021 Apr 23;11:630706. doi: 10.3389/fonc.2021.630706 (PMC8103545; doi:10.3389/fonc.2021.630706)
Supplement: Supplementary file 1 [file Table_1.docx]

| ID | coef | HR | HR.95L | HR.95H | p-value |
| --- | --- | --- | --- | --- | --- |
| IFNG | 0.544385 | 1.723549 | 1.374546 | 2.161164 | 2.41E-06 |
| KIR2DL4 | 0.486876 | 1.627225 | 1.017342 | 2.602725 | 0.042183 |
| TUBB3 | 0.465755 | 1.593216 | 1.086728 | 2.335762 | 0.017031 |
| NOD2 | 0.458007 | 1.58092 | 1.125962 | 2.219708 | 0.008166 |
| UCN | 0.417576 | 1.518276 | 1.238659 | 1.861016 | 5.80E-05 |
| IRF9 | 0.39729 | 1.487787 | 1.204609 | 1.837535 | 0.000226 |
| LTB4R2 | 0.367736 | 1.44446 | 0.933644 | 2.234754 | 0.098617 |
| ULBP2 | 0.287226 | 1.332726 | 1.11082 | 1.598961 | 0.001995 |
| PDIA2 | 0.260822 | 1.297996 | 1.115355 | 1.510546 | 0.000749 |
| FCGR2B | 0.259348 | 1.296085 | 1.089372 | 1.542023 | 0.003438 |
| HCST | 0.03156 | 1.032063 | 0.999155 | 1.066054 | 0.005628 |

**The immune-related gene HCST as a novel biomarker for the diagnosis and prognosis of clear cell renal cell carcinoma**

**Supplementary table**

**Supplementary Table S1. 13 PDEIRGs in prognostic model.**
